# Production, detection, storage and release of spin currents

**DOI:** 10.3762/bjnano.6.75

**Published:** 2015-03-13

**Authors:** Michele Cini

**Affiliations:** 1 Dipartimento di Fisica and Istituto Nazionale di Fisica Nucleare, Università di Roma Tor Vergata, Via della Ricerca Scientifica 1, I-00133 Rome, Italy

**Keywords:** quantum pumping, quantum transport, spin current

## Abstract

**Background:** Quantum rings connected to ballistic circuits couple strongly to external magnetic fields if the connection is not symmetric. Moreover, properly connected rings can be used to pump currents in the wires giving raise to a number of interesting new phenomena. At half filling using a time-dependent magnetic field in the plane of the ring one can pump a pure spin current, excited by the the spin–orbit interaction in the ring.

**Results:** Such a magnetic current is even under time reversal and produces an electric field instead of the usual magnetic field. Numerical simulations show that one can use magnetizable bodies as storage units to concentrate and save the magnetization in much the same way as capacitors operating with charge currents store electric charge. The polarization obtained in this way can then be used on command to produce spin currents in a wire. These currents show interesting oscillations while the storage units exchange their polarizations.

**Conclusion:** The magnetic production of spin currents can be a useful alternative to optical excitation and electric field methods.

## Introduction

The time-honored field of quantum transport has been evolving in the past 15 years in such a way that spin–orbit interaction effects and spin currents have become one of the main directions, also in view of promising applications to the new field of spintronics. An excellent review has been provided by Žutić and co-workers [1]. In recent years there has been in the literature a growing interest in the relation between magnetic and transport properties of materials [2]. On the other hand, other promising themes of research on spin currents remain largely to be explored.

One is the spin version of quantum pumping – something that is not possible classically. A seminal paper by Thouless [3] pointed out that quantum mechanics gives the possibility of propagating a charge current in a circuit without an applied bias. It became clear later that quantum pumping obtained by varying one parameter in the Hamiltonian requires a nonlinear dependence of the current on the dynamics. Romeo and Citro [4] have discussed memory and pumping effects in oscillators coupled to parasitic nonlinear dynamics. These ideas must be extended to spin currents.

As another example, systems containing rings and having a nontrivial topology are another important direction of research that has been given relatively little attention to date in this context. Nevertheless, it is clear that such systems will soon occupy the front of stage when the focus will shift on the mechanical effects of magnetic fields in nanoscopic systems. The interest in quantum ring properties is rather fundamental since they show striking deviances from classical properties [5] and also because of the growing role of topological effects in this field.

**Figure 1:**
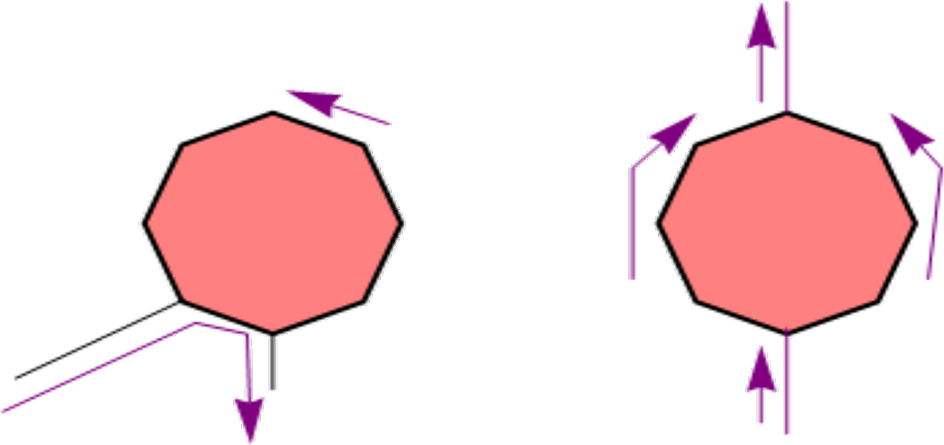
Left: Lateral connection of a quantum ring (*N*_ring_ = 8) to external wires (any even number of sides greater or equal than four can be considered, since the graph is bipartite, see below.) The flow of a current in the external wire selects a chirality in the ring and produces a magnetic moment. Right: a symmetric connection to the external wire. No magnetic moment is produced.

The present paper belongs to a series devoted to the magnetic properties of quantum rings linked tangentially to ballistic circuits. The geometry is explained in Figure 1. If the ring-wire connection is asymmetric, a current in the external circuit selects a chirality in the ring and produces a magnetic moment. As we shall see, the reverse is also true, namely, a chiral current in the ring can pump charge in the wire. By the same token, a symmetrically connected quantum ring inserted in a circuit cannot choose a chirality and has zero magnetic moment when a current flows through it. A symmetric connection (Figure 1 right) is unfavorable for quantum pumping. This is why a maximally asymmetrical connection is relevant in this respect. I call this geometry a laterally connected ring (see Figure 1 left).

The effects described in the present paper are purely quantum mechanical, because spins are involved and also because pumping phenomena are classically forbidden. Even spinless models with laterally connected rings give raise to pumping [6]. One method for pumping a charge current while leaving the ring neutral is based on the introduction of integer numbers of fluxons. The magnetic moment of the ring also deviates strongly from classical theory [5]. While one-parameter pumping is forbidden in a linear system [7], quantum effects produce nonlinearity and pumping. In the adiabatic limit there is no pumping at all, as shown in a beautiful general analysis by Avron, Raveh and Zur [8]. However, if a flux of the order of a flux quantum is inserted in a time of the order of the electron hopping time, an electron is pumped in response of an inserted flux of the order of a fluxon [6]. The present treatment neglects interactions. However, the above phenomena were studied within the Luttinger liquid [9] approach. It was found that the pumping phenomena persist unhindered in the presence of the interactions [10].

In [11], it was shown that laterally connected rings have peculiar properties for quantum pumping. One can pump spin-polarized currents into the wires by using rotating magnetic fields or letting the ring rotate around the wire. This method works without any need for a spin–orbit interaction, and without stringent requirements about the conduction band filling. Besides, they can be used to pump spin, rather than just charge. In principle, quantum mechanics allows us to build a device to achieve that in more than one way. One can also build a ring device that can produce a pure spin current, that is, a magnetic current without any charge current associated to it [12].

In the present paper, I concentrate on the last thought experiment in order to better clarify the physical meaning of the spin current obtained in this way. This can be done by more thought experiments. The results suggest that one can transfer magnetization between two distant bodies directly through a lead, without moving any charges. Also, one can we detect the spin currents by the field they produce around the wire. Moreover, one can use a spin current to magnetize bodies and later use the magnetized bodies to excite a pure spin current in a wire. The experimental realization of a magnetic current could also be useful for the ongoing research of an electric dipole moment of the electron. Moreover such theoretical ideas, once realized in a practical device, would offer new strategies to attack the problems connected to spintronic applications.

Alternative mechanisms for generating pure spin currents have been proposed. Bhat and Sipe [13] proposed using circularly propagating light beams to excite polarized spin currents. Brataas and coworkers [14] predicted that precessing ferromagnets inject spin currents in semiconductors. The spin Hall effect [15–16] uses an electric field. The novel mechanism based on laterally connected rings is driven by a time-dependent magnetic field.

## Geometry and dynamics of spin current generation

In this Section, I recall the Hamiltonian *H*_prod_, the same as in [12], which, in the half filling case, describes the magnetic production of the spin current based on a quantum pumping effect in the absence of an external bias. Below, *H*_prod_ will be complemented with other terms to allow for spin current storage and release.

[1]
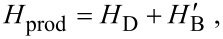


where *H*_D_ is the device Hamiltonian and *H*′_B_ the in-plane magnetic term. Here,

[2]
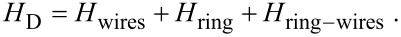


The polygonal ring, with an even number *N*_ring_ of sides, which ensure a bipartite lattice, is represented by

[3]
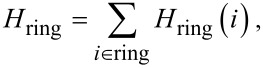


where, with the identification of ring site *N*_ring_ + 1 with site 1, we may write

[4]
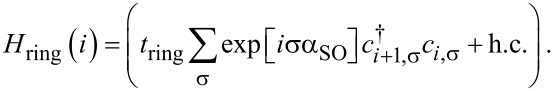


Here α_SO_ is a phase due to the spin–orbit interaction [17], and can be of order unity or smaller.

Figure 1 left shows the geometry for a ring of *N*_ring_ = 8 vertices, but any polygon with even *N*_ring_ grants a pure spin current. The vertices of the polygon represent sites and are acted upon by the spin–orbit interaction and by the external magnetic field *B*(*t*). The side length may be taken to be of order of a few angstroms. All sites are connected to the first neighbors by spin-diagonal matrix elements.

The Hamiltonian for the left and right wires is a standard tight-binding model

[5]
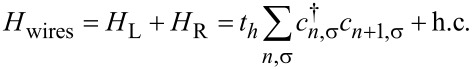


Finally, the ring–wires contacts are modeled in *H*_ring−wires_ whereby the ring has a nearest neighbor connection to the leads via a tunneling Hamiltonian with hopping matrix elements *t_lr_* in the obvious way.

The magnetic interaction due to *B*(*t*) acts exclusively on spin and is:

[6]
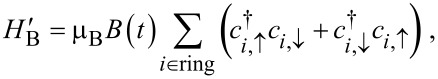


with the Bohr magneton μ_B_ = 5.79375 · 10^−5^ eV/T. The time-dependent field lies in the plane of the ring and has no flux through it. However, it couples to the electron spins in the ring, where the magnetic spin-flip combined with the spin–orbit interaction excites the spin current that is pumped to the external wires. Finally, I note that in this formulation there are no mean-free-path effects. This is appropriate to systems that are small compared to the mean free path. In such cases the transport is ballistic.

### Numerical evolution and calculation of the currents

For *t* < 0, *B* = 0 and the system is in thermal equilibrium with a spin-independent chemical potential *E*_F_ at temperature *T*. The natural time unit for this problem is 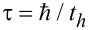. In the code the Hamiltonian is constant during time slices of 0.2τ and jumps to the next value at the end. In this way, the many-electron Schrödinger equation is integrated by a succession of sudden approximations.

Taking the spin quantization axis along *z* (orthogonal to the plane of the ring), the number current operator may be written as:

[7]
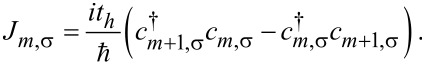


I calculate the time-dependent number current by the partition-free approach [18], namely,

[8]
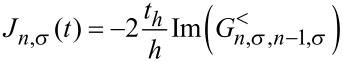


where, in terms of the retarded function 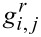,

[9]
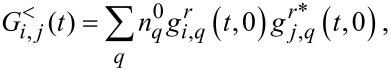


where *q* runs over the ground state spin-orbitals for *B* = 0 and 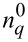 is the Fermi function.

In the numerical calculations below I take *t_h_* = *t*_ring_ = *t*_lr_ = 1 eV as a reasonable order-of-magnitude, and the temperature is absolute zero. My codes calculate number currents taking *t_h_* = 1. If this is interpreted to mean that *t_h_* = 1 eV, which corresponds to the frequency 2.42 · 10^14^ s^−1^, a current *J* = 1 from the code means 2.42 · 10^14^ electrons per second, which corresponds to a charge current of 3.87 · 10^−5^ A. We also need a characteristic magnetic field. Recalling that 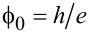 ≈ 4.134 10^−15^ in MKSA units, we introduce the magnetic field 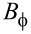 such that 
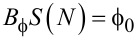
 where


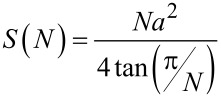


is the ring area. Thus,


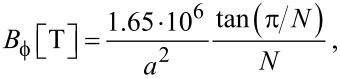


with *a* in angstroms.

In the present geometry both spin directions orthogonal to the ring are treated on the same footing. The spin symmetry is broken by α_SO_ which provides a driving force. For any time dependence of 
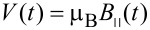
 the charge current vanishes identically at half filling and a pure spin current obtains.

A good analytic understanding of the purely spin current, of its non-adiabatic character, and of its topological origin was achieved in [12]. The absence of a charge current can be deduced from the invariance of the problem under a canonical transformation that exchanges electrons with holes, spin-up with spin-down and changes sign to one of the sublattices. The exact analytical results of [12] need not be repeated here. However, I note that the proof of the absence of a charge current can be extended to finite temperatures simply by replacing the ground state average used there by a Grand-Canonical one.

Numerical results obtained with an odd *N*_ring_ show a partial spin polarization, in line with the fact that they do not correspond to bipartite lattices. The extra atom produces a charge current that becomes small when the ring gets large. Below we consider even *N*_ring_.

### Field produced by a stationary spin current

A line of dipole moments polarized along *z* and extending along the *x*-axis would be described by the magnetic moment linear density

[10]
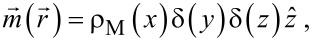


where 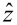 is the unit vector along z, ρ_M_(*x*) is a magnetization density and δ(*x*) is Dirac’s delta. This will produce a vector potential that we can write in the form

[11]
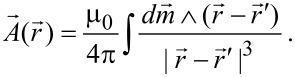


Here μ_0_ is the vacuum susceptibility. For a constant distribution ρ_M_ the integral yields, in components,

[12]
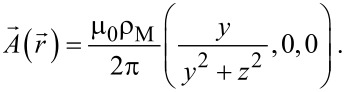


If the line of dipole moments moves along the *x*-axis with speed *u*, in the stationary system the dipole density is 
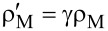
 with γ = 1/[1 − (*u*^2^/*c*^2^)]^(1/2)^, while the four-potential is found by a Lorentz transformation. It reads 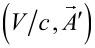 where 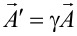, and the scalar potential is

[13]
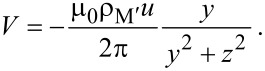


One can represent a time-independent spin-current semiclassically by two superimposed lines of opposite polarization moving with opposite speeds. Their vector potentials cancel each other out and one is left with a scalar potential

[14]
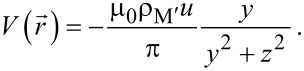


This shows a duality between the electric current producing a magnetic field and the magnetic current producing an electric field. This duality has already been pointed out [19] in a different model, but in view of the possibility to obtain pure spin currents in nanoscopic objects (where due to the nanoscopic dimensions, intense fields prevail at short distances from the wire) it acquires an extra significance, as we shall see. To begin with, although these treatments are semiclassical and approximate, the vanishing of the magnetic field is exact, since the pure spin current is even under the time reversal operator *T* while the magnetic field is odd.

In polar coordinates, with *y* = *r* cos(θ), *z* = *r* sin(θ), the electric field components are given by:

[15]
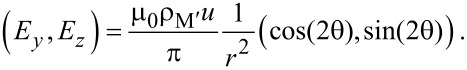


**Figure 2:**
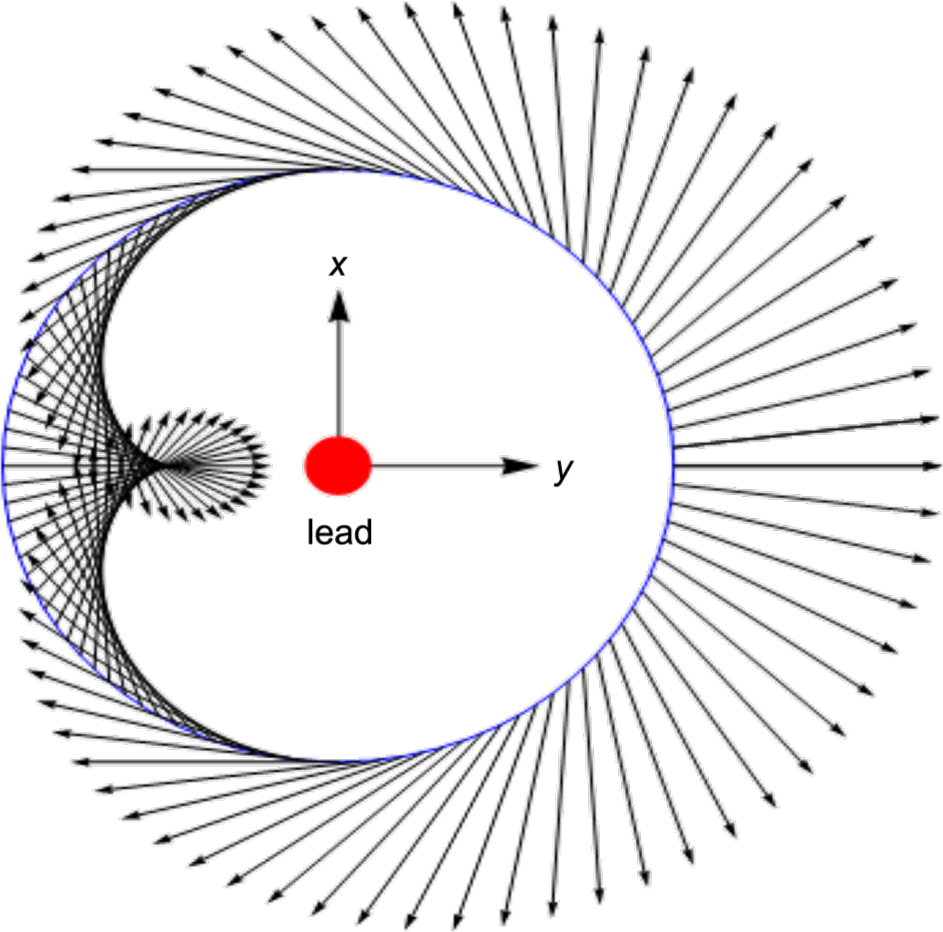
Pattern in the *y*–*z*-plane of the electric field produced by a pure spin current flowing in the lead, parallel to the *x*-axis, represented here by the disk at the origin. The magnetic field is along the *y*-axis.

The pattern is shown in Figure 2. Note that all the black arrows represent the direction of 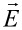 at a fixed distance from the lead (central disk) and start from the light blue circle. However, if one starts from the *y*-axis (direction of 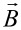) and makes an angle of π, the direction of 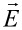 rotates by 2π.

This field is without divergence and rotation outside the singular line of the lead. No charge distribution could yield this pattern. This field symmetry could in principle be used to detect and measure the spin current, even if it were in combination with a normal charge current.

However, the experimental realization of a pure spin current would have a more fundamental significance, because of its time-reversal invariance. It is *T*-even, while an electric current is trivially *T*-odd. Particle physicists are trying to measure, or put constraints on, the electric dipole moment of the electron [20] by using strong fields in molecular [21] or solid-state measurements [22]. Formally, the electron anomalous magnetic moment is describes in Dirac’s theory by a term proportional to *F*_μν_σ^μν^ψ, where ψ is Dirac’s spinor, *F*_μν_ and σ^μν^ are the electromagnetic and spin tensors, respectively [23]. A term in *F*_μν_σ^μν^γ_5_ψ would yield the effects of an electric dipole moment. However, this would be odd under time-reversal, and it was originally discarded for this reason. No experimental fact supports its existence. Now, however, such a term would be most wanted by people looking for new physics, and is predicted by several theories trying to explain, e.g., the excess of matter over antimatter in the universe. If such a term exists, it should produce a *T*-odd term in the pure spin current and a magnetic field that could be sought for. A recent [20] upper limit for the electron dipole moment is 8.3 · 10^−17^μ*_B_*. Can we do better in this way? Since in the present approach the time dependence *B*(*t*) is arbitrary one can also consider a time-dependent spin current radiating electromagnetic waves. The quantitative estimate of the accuracy of this method is beyond the scope of the present paper and is a rather demanding task. Detailed calculations are under way.

### Storage, detection and delayed release of the spin current

The possibility of exciting a pure spin current suggests that a magnetization transfer without charging of the kind outlined in the Introduction should be feasible by using a laterally connected polygonal ring and a magnetic field in the same plane as the ring. It should be possible to store and possibly concentrate the spin current in the form of a long-lived static polarization of a storage electron gas for later use. Actually the polarization would be permanent in the absence of the spin–orbit interaction in the reservoirs. In materials with low atomic numbers the polarization could be long-lived enough to make interesting experiments on it and maybe even technological applications. A measurement of the magnetic moment of the storage bodies would also serve to detect and measure the time integral of the spin current.

Here, I wish to present computer experiments aimed at this target, in which the storage bodies are cubes of various sizes. The total Hamiltonian is given as:

[16]
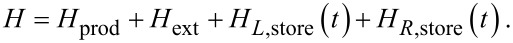


Here, *H*_prod_ is the above described Hamiltonian with a 6-site ring. The field parallel to the *y*-axis was taken to grow linearly form 0 to 32 T at time *t* = 80 τ. I recall that time is measured in units of 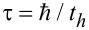. The spin–orbit constant was taken to be α_SO_ = 1. The additional terms in Equation 16 describe the left and right storage units *H_L_*_,store_(*t*), *H_R_*_,store_(*t*) and the external wire *H*_ext_ of the same form as Equation 5 connecting the identical storage unit.

Below, the storage units will be taken to be 2 × 2 × 2 or 3 × 3 × 3 cubic clusters of sites, which are bipartite. One must choose *H_L_*_,store_(*t*) and *H_R_*_,store_(*t*) such that the whole graph is bipartite, and the storage units will be half filled initially and for all times. Here *H_L_*_,store_(*t*) and *H_R_*_,store_(*t*) also contain the connections to the external wire and to the respective wires to the ring, and these depend on the time according to the following pattern.

Initially, the whole system is in equilibrium in the absence of external fields. Then the direct cube–cube connection is removed and the external field in the plane of the ring starts to excite a spin current that will polarize the cubes in the opposite way. Indeed, the numerical results confirm that a pure spin current flows in the wires from the ring to the cubes, and its polarization axis is in the *y*–*z*-plane. Then, the cubes are isolated and the spin current is stored in them as a constant magnetization. Finally, the direct connection through a 6-site lead is established. Then, a spin current is excited in the connecting lead while the up and down spin populations in both rings oscillate.

The sum of spin-up and spin-down populations in each cube was constant and the two uncharged rings are oppositely polarized. The pattern was similar when the spin quantization axis was taken along *y* or along *z*. These oscillations look similar to those already observed in spin-injection experiments, described by Albert Fert in his Nobel lecture [24]. Those observed thus far are in the microwave range, while in the present approach the characteristic frequency is proportional to the hopping integral. This suggests that higher frequencies than those reported in the literature could be attained.

**Figure 3:**
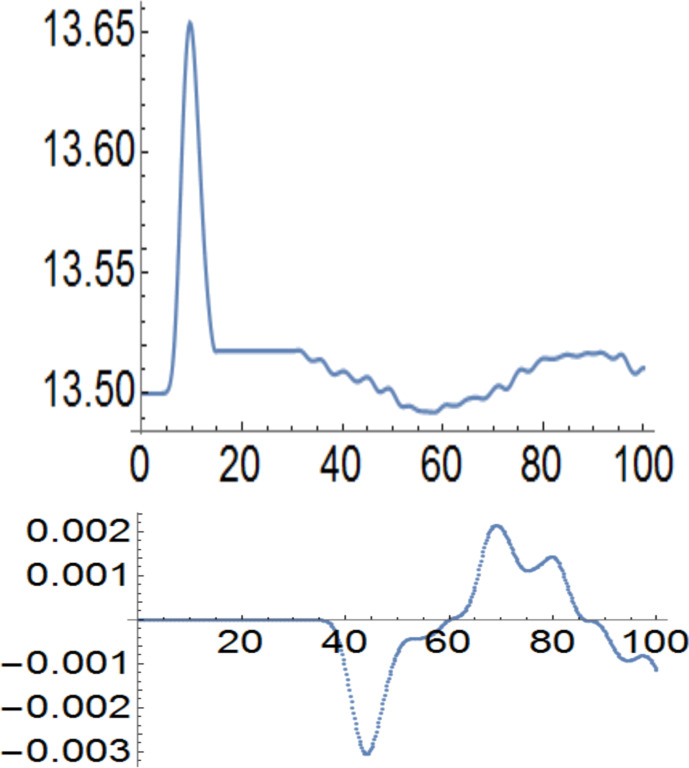
Upper panel: up-spin population of the left storage cube versus time measured in units of τ. Lower panel: spin-up current in the wire connecting the two 27-site cubes, where the hopping integral is *t*_ext_ = 0.25 *t_h_*.

**Figure 4:**
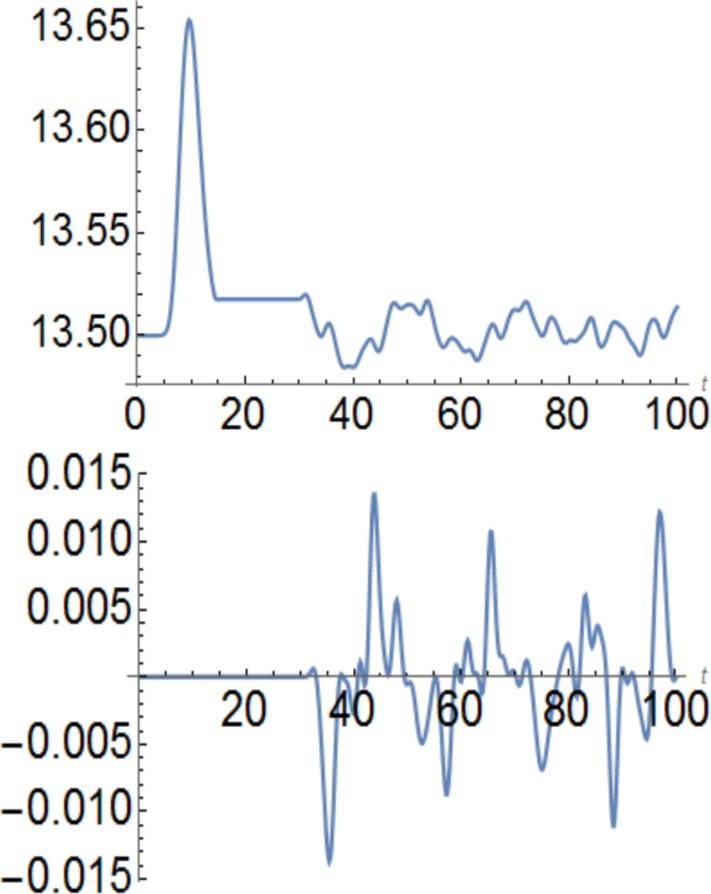
Upper panel: up-spin population on the left storage cube versus time measured in units of τ. Lower panel: spin-up current in the wire connecting the two 27-site cubes, where the hopping integral is *t*_ext_ = 0.75 *t_h_*.

In Figure 3 and Figure 4, the storage rings have 27 sites, the ring-cubes connections have 13 sites and the cube–cube connection has 6 sites. The populations start from half filling, that is, 13.5 electrons per spin. Then the left cube receives an up-polarization and the polarization of the right ring is opposite. One could engineer the timing of the process and the size of the storage units if the aim is to maximize the polarization. From *t*_1_ = 15τ to *t*_2_ = 30τ the cubes are isolated and polarization is constant, as it should be. In both cases, the polarization transfer without charging is quite substantial. The cubes remain strictly neutral like the rest of the system. At *t*_2_ the cubes are connected through a wire which has a hopping integral *t*_ext_ = 0.25 *t_h_* in Figure 3 and *t*_ext_ = 0.75 *t_h_* in Figure 4. The upper panels show the evolution of the spin-up occupation, while the lower panel shows the spin-oscillating currents in the wires connecting the reservoirs. The spin-down currents are opposite. The current oscillates because the reservoirs exchange their polarizations. In Figure 4, the 6-site connection between the reservoirs has a larger hopping constant and produces a stronger and faster spin polarization exchange.

**Figure 5:**
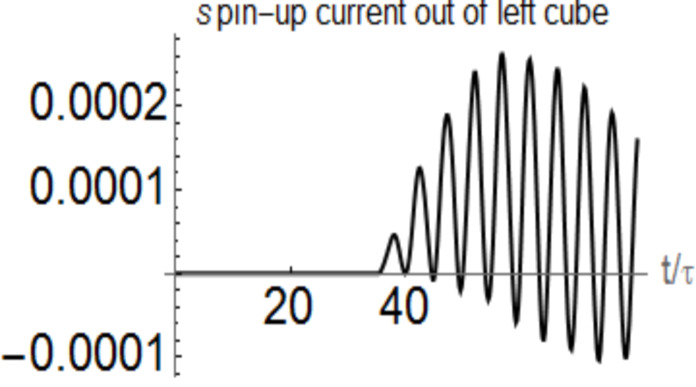
Spin-up current from the left storage cube in the cube–cube connection, when both storage cubes have eight sites. The hopping integral in the cube–cube wire is *t*_ext_ = 0.05 *t_h_*. The spin-down current is opposite.

The spin-up current in the cube–cube connection in a similar numerical experiment with 8-site cubes is shown in Figure 5. It starts at time *t* = 30τ when the cubes are connected via a 6-sites wire and shows oscillations. It should be interesting to observe the spectrum and polarization of electromagnetic waves emitted by these oscillating magnetic currents. This would be another way to detect the spin current.

## Conclusion

I presented a theoretical study of tight-binding model devices consisting of a ring laterally connected to a wire and designed to produce spin polarized currents at half filling. A tangent time-dependent magnetic field in the plane of the ring can be used to pump a magnetic, i.e., purely spin current, excited by the spin–orbit interaction in the ring. This behavior is analytically described in [12] and is found to be robust with respect to temperature and small deviations from half filling.

The main results of the present paper concern possible schemes to detect, store, gather, and then release magnetization. Suitable reservoirs or storage units have been shown to work with spin currents in analogy with capacitors for common charge currents. The spin polarization can be stored without charging the reservoir and then used on demand to produce pure spin currents in a wire. These currents show oscillations while the storage units exchange their polarizations and the intensity and amplitude of the oscillations can be modified by changing the conductivity of the link between the polarized units. The experimental realization of a magnetic current could also be relevant for the ongoing research of time-reversal violating new physics. Detailed calculations of such fields are under way.

The present model neglects electron–electron interactions, but it is physically reasonable that adding to the Hamiltonian a correlation term such as 
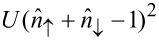
 would tend to reinforce the charge pinning effects described here. At any rate, in the Hartree approximation, it would change nothing since its average at half filling vanishes strictly during the evolution of the system as a consequence of the theorem of [12].

In the present work the currents were generated by the action of a magnetic field, without the need of an external bias. However, the results have implications on the general problem of quantum transport in ballistic, topologically non-trivial circuits. Compelling physical arguments based on thought experiments [5] suggest that the magnetic moment of a quantum ring is not obtained by substituting the quantum current in the classical formula [25]. The basic reason is that any measurement of the magnetic moment of the ring requires the measurement of a force exerted on the ring by a probe flux, which according to quantum mechanics has an influence on the current. Actually, rephrasing the conclusions of [5], the magnetic moment is given by the operator

[17]
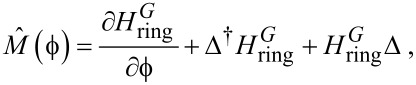


where 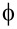 is the probe magnetic flux through the ring, 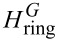 is the grand-canonical ring Hamiltonian referenced to the equilibrium chemical potential of the system and Δ = 
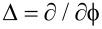
. As a consequence, when the circuit is biased by a small potential difference *V*_bias_ and a small current flows, 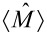 is found [5] to go with 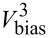 while one classically expects *M* ~ *V*_bias_ in a linear circuit. Indeed, at small *V*_bias_ the quantum effects favor a laminar current which is not coupled to the magnetic field. The main subject of the present work can also be considered a complementary way to study the quantum effects of [6] in the reversed situation when *V*_bias_ = 0 and it is the interaction of the ring with a magnetic field that produces a current in the external circuit.

The above theoretical effort leads to several intriguing possibilities from the viewpoint of basic research and possible applications and I hope that this will stimulate experimentalists to work on the magnetic properties of laterally connected quantum rings.
